# Norepinephrine versus angiotensin II in septic shock: effects on isolated kidney, heart and liver mitochondrial respiration

**DOI:** 10.1186/cc10803

**Published:** 2012-03-20

**Authors:** V Jeger, M Vuda, T Correa, J Takala, S Djafarzadeh, SM Jakob

**Affiliations:** 1 Inselspital University Hospital Bern, Switzerland

## Introduction

Mitochondrial dysfunction has been proposed to influence organ function and outcome in sepsis. Both vasopressor agents norepinephrine and angiotensin II can interfere with mitochondrial function. The aim of this study was to compare mitochondrial respiration after exposure of septic animals to either of these two drugs.

## Methods

In 16 anesthetized pigs, evolving septic shock after 12 hours of fecal peritonitis was randomly treated with either norepinephrine (0.8 ± 0.6 μg/kg/minute; mean ± SD) or angiotensin II (0.31 ± 0.37 μg/kg/minute; *n *= 8, each) and fluids for 48 hours. Organs were harvested at the end of the experiment, and mitochondria isolated by tissue homogenization and differential centrifugation. Mitochondrial oxygen consumption (VO_2_) was measured by high-resolution respirometry (Oroboros Instruments, Innsbruck, Austria). Groups were compared using Mann-Whitney U test. In addition, mitochondrial respiration was also compared to a similarly instrumented control group without fecal peritonitis (*n *= 8; Kruskal-Wallis test).

## Results

Achieved blood pressure levels and cardiac output were not different between the two septic groups, and both groups received the same amount of fluids (norepinephrine: 1.6 ± 0.5 ml/kg/hour, angiotensin II: 1.3 ± 0.8 ml/kg/hour; *P *= NS). Compared to controls, mitochondrial VO_2 _was not different in septic animals. The only difference between the two septic groups was higher renal Complex I, State 4 respiration in norepinephrine-treated (median (range): 309 (164 to 415) pmol/(second*mg)) versus angiotensin-II-treated animals (210 (89 to 273) pmol/(second*mg); *P *= 0.05).

## Conclusion

We found no significant effects of septic shock treated with either angiotensin II or norepinephrine and fluids on mitochondrial function, under similar hemodynamic conditions. Hepatic, renal and myocardial respiration of the measured mitochondrial complexes did not significantly differ between the two treatment groups, except for renal Complex I, State 4 respiration.

